# Abrocitinib and dupilumab bridging therapy for bullous pemphigoid with insufficient response to initial corticosteroid therapy: a case report

**DOI:** 10.3389/fimmu.2026.1906365

**Published:** 2026-08-24

**Authors:** Hongchan Zhang, Linqi Li, Susu Zhao, Hao Wu, Jianwei Zhu

**Affiliations:** 1 Department of Dermatology, Longyou County Traditional Chinese Medicine Hospital, Longyou, Zhejiang, China; 2 Department of Dermatology, Quzhou Traditional Chinese Medicine (TCM) Hospital at the Junction of Four Provinces Affiliated to Zhejiang Chinese Medical University, Quzhou, China

**Keywords:** abrocitinib, bridging targeted therapy, bullous pemphigoid, dupilumab, JAK1 inhibitor, pruritus, type 2 inflammation

## Abstract

Bullous pemphigoid (BP) is a chronic autoimmune blistering disease predominantly affecting the elderly, with conventional immunosuppressive treatments often limited by insufficient efficacy and severe adverse effects. Emerging targeted therapies offer new opportunities, yet optimal treatment strategies remain to be defined. Herein, we report a case of BP in a 69-year-old woman with insufficient response to initial systemic corticosteroid therapy, successfully managed with a bridging targeted approach using abrocitinib and dupilumab. Initiation of abrocitinib (200 mg daily) resulted in rapid pruritus relief within 24 hours and cessation of new blister formation within 6 days. Symptom relapse and reinduction upon dose reduction and restoration suggested a dose-dependent therapeutic effect of abrocitinib. Dupilumab was subsequently introduced for long-term disease control, while abrocitinib was gradually tapered off. After more than six months of targeted therapy, sustained complete remission was achieved, with a favorable safety profile. Objective disease activity improved in parallel with clinical remission, with BPDAI decreasing from 120 at baseline to 0, pruritus NRS from 9 to 0, and peripheral eosinophil counts returning to the normal range during follow-up. Mechanistically, abrocitinib may rapidly suppress pruritus and inflammation through JAK1-dependent blockade of IL-4, IL-13, and IL-31 signaling. In contrast, dupilumab provides sustained disease control by inhibiting IL-4/IL-13-mediated Th2 responses, reducing eosinophilic inflammation and autoantibody production. This case highlights a complementary therapeutic strategy integrating rapid disease control with long-term immune modulation, suggesting that our bridging therapy may represent a rational and effective approach for patients with bullous pemphigoid with insufficient response to initial corticosteroid therapy, warranting further investigation in controlled studies.

## Introduction

Bullous pemphigoid (BP) is a chronic autoimmune blistering disease predominantly affecting the elderly, especially in patients older than 70 years of age (1). The cumulative incidence of BP is estimated as 8.2 per million people, whereas the incidence rate is 34.2 per million person-years (2). It is characterized by tense blisters and erosions arising on erythematous, urticarial, or normal skin. The pathogenesis is still unclear. The development of BP is linked to the generation of autoantibodies that target hemidesmosomal proteins BP180 (collagen XVII) and BP230, which are located at the basement membrane zone and are essential for the dermo-epidermal junction (DEJ) (3). Furthermore, numerous studies have implicated type 2 cytokines, including IL-4 and IL-13, in contributing to the pathogenesis of BP (4). Pruritus is also a common major complaint of BP patients. IL-31 is a key pruritogenic factor, and the expression of its receptors (IL-31RA and OSMRβ) correlates with the severity of pruritus (5). The primary treatment modalities for BP include glucocorticoids and immunosuppressive agents. However, some patients are refractory to conventional therapy or are prone to side effects, highlighting the need for alternative, targeted approaches.

The advent of novel targeted therapies has opened new avenues for managing refractory BP. Existing studies and case reports have demonstrated that Janus kinase inhibitors achieve favorable therapeutic outcomes in blistering skin diseases through the interruption of multiple cytokine signaling cascades (6–10). Abrocitinib, a highly selective JAK1 inhibitor, rapidly exerts antipruritic and anti-inflammatory effects by blocking IL-4, IL-13, and IL-31 signaling (11). Dupilumab, an IL-4Rα antagonist that simultaneously inhibits both the IL-4 and IL-13 pathways, suppresses Th2-driven autoantibody production and has recently been approved by the FDA for moderate-to-severe bullous pemphigoid (12). Although single-agent targeted therapy has shown promise, the combination of abrocitinib and dupilumab has not been previously reported in patients with bullous pemphigoid showing an insufficient or refractoy response to corticosteroid therapy. This article explores the complementary advantages of “rapid induction remission” and “long-term safe maintenance” achieved through bridging therapy with abrocitinib and dupilumab in a patient with bullous pemphigoid who had an inadequate response to initial corticosteroid therapy, and analyzes the underlying immunological mechanisms, and provides a reference for future combination therapies.

## Case presentation

A 69-year-old woman weighing 64 kg presented with a 1-month history of widespread pruritic erythematous patches and tiny blisters on her trunk, limbs, head, and face. The eruptions began 2 days after she underwent closed reduction and internal fixation for her left ankle fracture. She was initially treated at a local hospital with oral methylprednisolone (8 mg three times daily) for presumed eczema. However, the lesions and pruritus persisted, prompting referral to our dermatology clinic.

**Figure 1:**
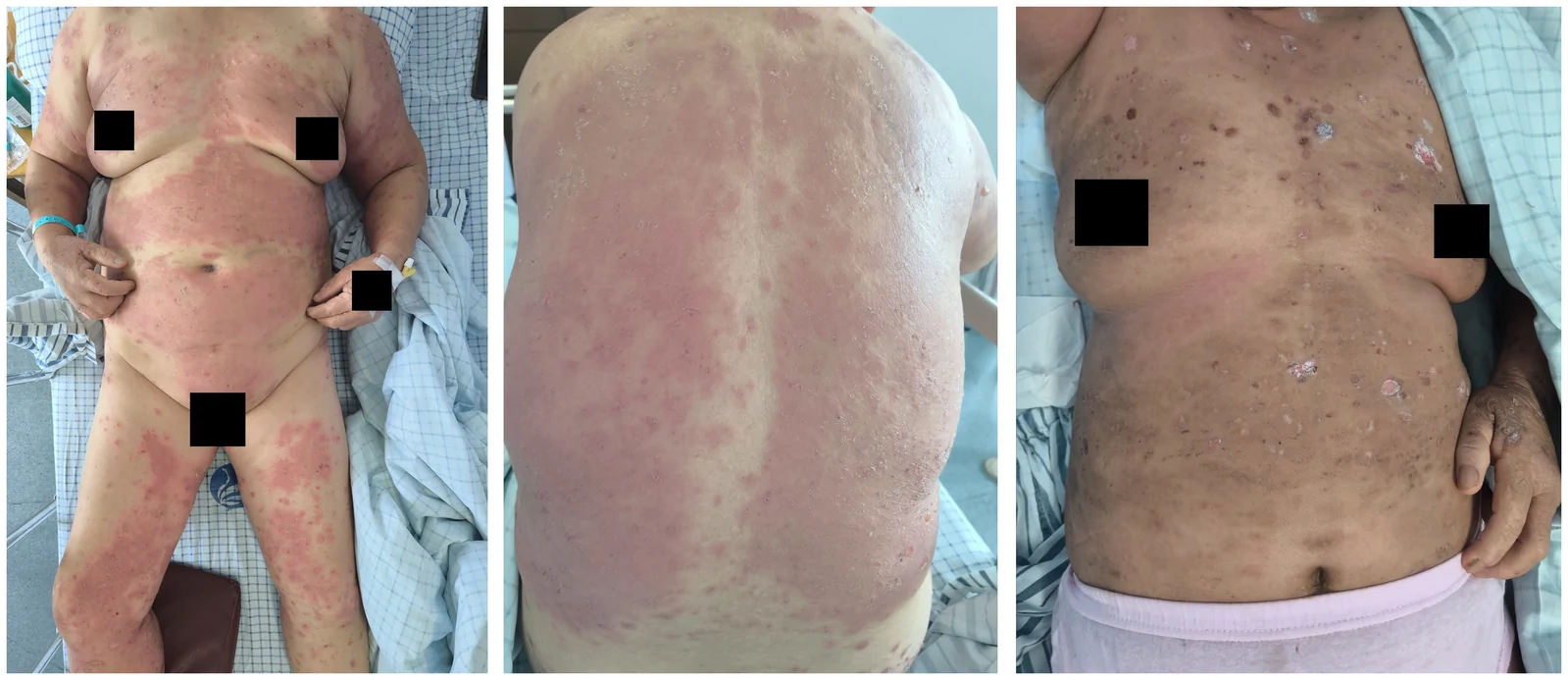
Imaging presentation of the patient.

**Figure 2:**
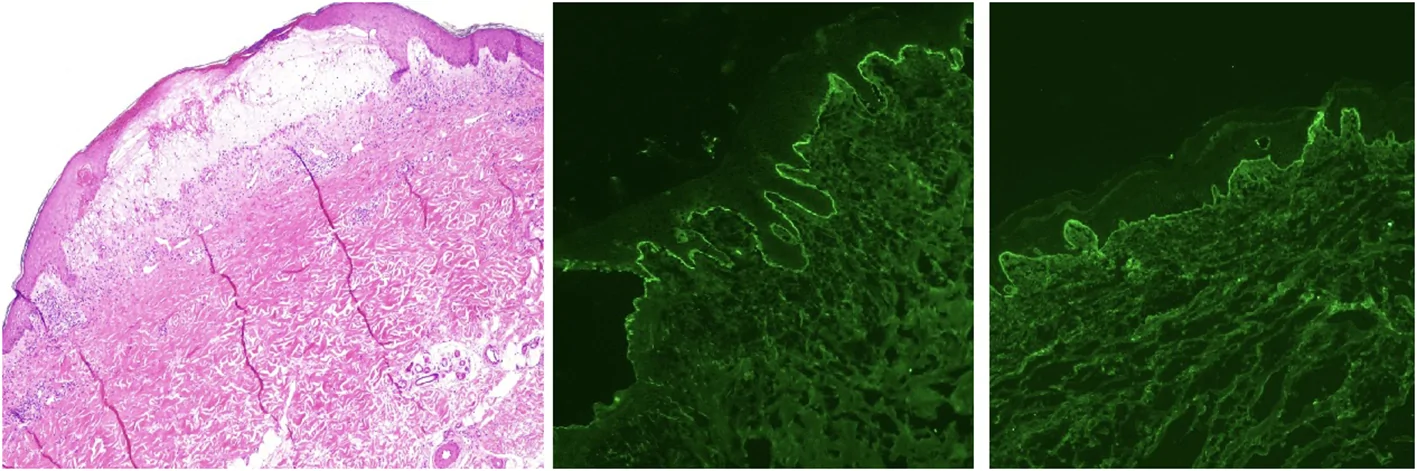
Examination.

Physical examination revealed extensive erythematous patches on the trunk and extremities, with scattered tense blisters measuring 1-5 mm in diameter on an erythematous base (**Figures 1A, B**). No mucosal involvement was noted. Nikolsky’s sign was negative. To establish a definitive diagnosis, a skin biopsy was performed. Histopathological examination showed a subepidermal blister with a prominent eosinophilic infiltrate, and direct immunofluorescence demonstrated linear deposits of IgG and C3 along the dermoepidermal junction (**Figures 2A–C**). Serum anti-BP180 antibody level was elevated at 372 AU/mL (reference range, < 20 AU/mL), confirming the diagnosis of bullous pemphigoid.

Initial treatment with intravenous methylprednisolone sodium succinate (40 mg once daily, 0.63mg/kg/day) combined with topical halometasone and triclosan cream was administered for 5 days. Because the disease remained active, the methylprednisolone dose was increased to 80 mg once daily (1.25mg/kg/day) for the next 5 days. However, clinical improvement remained insufficient despite 10 days of systemic corticosteroid therapy. Considering the patient’s history of hypertension, recent history of fracture surgery, and poor response to glucocorticoids, we decided to seek other more targeted therapies. Before initiation of abrocitinib, baseline safety assessments, including complete blood count, liver and renal function tests, lipid profile, and screening for tuberculosis and hepatitis, revealed no contraindications to treatment. Cardiovascular and thromboembolic risks were also assessed and considered acceptable. After detailed discussion with the patient and her family regarding potential risks and benefits, intravenous methylprednisolone was discontinued abruptly rather than gradually tapered, and topical halometasone was also discontinued. Abrocitinib (200 mg once daily) was then initiated without any other anti-inflammatory medications.

Following initiation of abrocitinib, her pruritus was remarkably relieved within 24 hours. The patient developed gastrointestinal symptoms, including nausea and vomiting, which are recognized adverse reactions of abrocitinib, and these symptoms were successfully managed with supportive care. On the third day of taking abrocitinib, the patient developed a fever, and laboratory tests revealed her eosinophil count was 4.94×10^9^/L, approximately 10 times higher than the upper limit of normal ((0.02-0.52) ×10^9^/L), together with increased neutrophils (13.2×10^9^/L). Her serum procalcitonin was normal and blood culture was negative. The fever resolved the following day. After 6 days of abrocitinib therapy, her pruritus was significantly reduced and no new blisters developed. Objective assessments also demonstrated early improvement, with the BPDAI decreasing from 120 to 96 and the pruritus NRS from 9 to 4 (**Table 1**).

**Table 1:** Longitudinal clinical course and objective disease assessment during therapy.

Time point	Clinical response	BPDAI	NRS	Therapy
Pre-abrocitinib	Severe pruritus, widespread erythematous plaques and blisters	120	9	Initial treatment with intravenous methylprednisolone sodium succinate (0.63mg/kg/day for 5days and 1.25mg/kg/day for 5days) combined with topical halometasone and triclosan cream
1 week post-abrocitinib	Pruritus and blisters remarkably improved, disease controlled	96	4	abrocitinib 200mg QD
Dose halved	Severe pruritus, explosion of numerous bullae	157	10	abrocitinib 100mg QD
Dose re-escalated	Progressive improvement, no new blisters, existing lesions drying	59	3	abrocitinib 100 mg BID + thalidomide (50 mg twice daily), tripterygium wilfordii glycosides (20 mg three times daily), promethazine (25 mg nightly), and ebastine (20 mg once daily)
2 week post-dupilumab	Lesions completely controlled, no new blisters	20	1	abrocitinib 100 mg BID + dupilumab 600 mg loading dose followed by 300 mg Q2W + thalidomide (50 mg twice daily), tripterygium wilfordii glycosides (20 mg three times daily), promethazine (25 mg nightly), and ebastine (20 mg once daily)
2 week later	Stable remission	11	1	abrocitinib 100mg QD +dupilumab 300 mg Q2W
1 month later	Stable remission	3	0	abrocitinib 100mg QD +dupilumab 300 mg Q2W
4 month later	Complete remission	0	0	dupilumab 300 mg Q2W

BPDAI, Bullous Pemphigoid Disease Area Index; NRS, Numerical Rating Scale.

**Figure 3:**
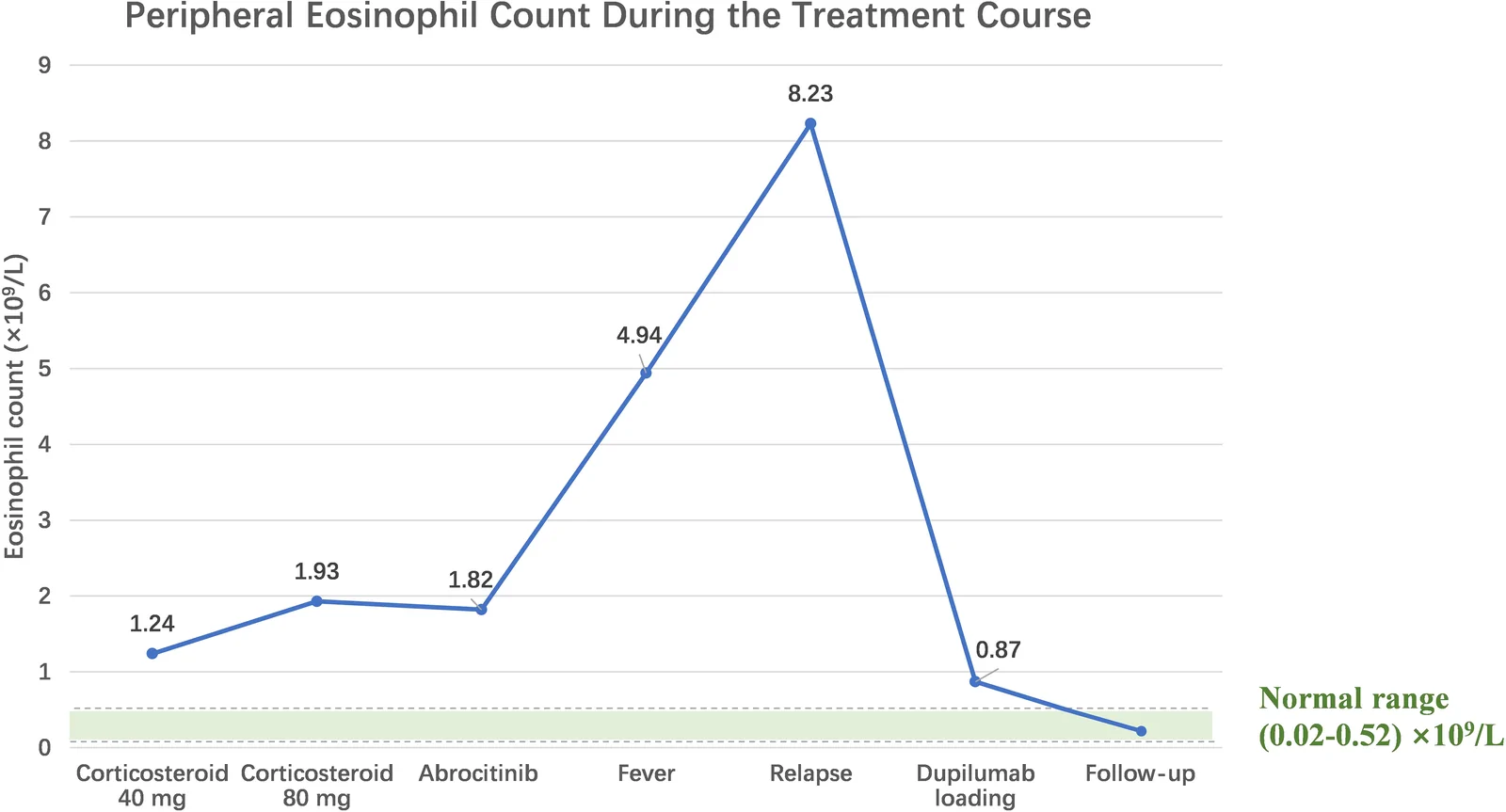
Peripheral eosinophil count during the treatment course.

Given the favorable response, we attempted to taper abrocitinib to 100 mg once daily to evaluate sustained therapeutic efficacy. However, the next day, the patient experienced a recurrence of severe pruritus accompanied by multiple newly developed and larger bullae, indicating a strong disease relapse. This deterioration was paralleled by objective worsening of disease activity, with the BPDAI increasing from 96 to 157, the pruritus NRS rising from 4 to 10, and the peripheral eosinophil count reaching a peak of 8.23×10^9^/L (**Table 1**; **Figure 3**). The abrocitinib dose was therefore restored to 200 mg/day, administered as two divided doses of 100 mg at noon and in the evening to improve gastrointestinal tolerability while maintaining the same total daily dose. Adjunctive therapies, including thalidomide (50 mg twice daily), tripterygium wilfordii glycosides (20 mg three times daily), promethazine (25 mg nightly), and ebastine (20 mg once daily) were also initiated because of the abrupt worsening of disease activity.

**Figure 4:**
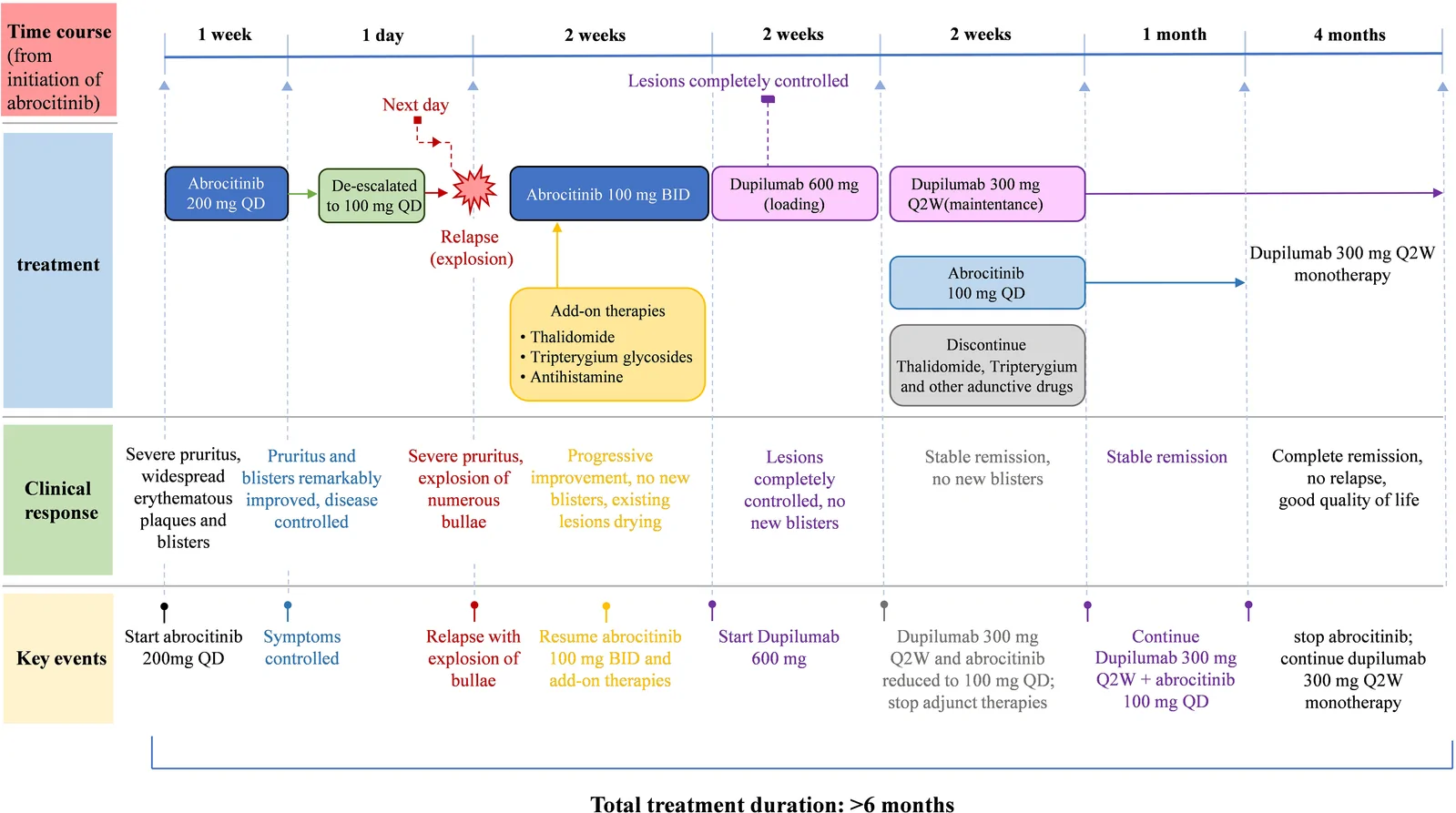
Clinical timeline of the patient.

After 2 weeks of this combination therapy, her pruritus had resolved completely; no new blisters developed, and most existing lesions had crusted (**Figure 1C**). Dupilumab was then initiated at a loading dose of 600 mg subcutaneously while the concomitant medications were maintained. 2 weeks later, dupilumab was continued at 300 mg every 2 weeks. At the same time, the abrocitinib dose was reduced to 100 mg once daily, and the adjunctive medications, including thalidomide, Tripterygium wilfordii glycosides, promethazine, and ebastine, were all discontinued. The patient continued treatment with dupilumab (300 mg every 2 weeks) plus abrocitinib (100 mg once daily) for another 1 month. Abrocitinib was then discontinued, and dupilumab monotherapy was then continued at 300 mg every 2 weeks for additional 4 months without concomitant topical corticosteroids or other systemic immunosuppressive agents (**Figure 4**). Routine clinical and laboratory surveillance during treatment, together with repeated venous thromboembolism risk evaluations, showed no serious treatment-emergent adverse events or thromboembolic events. Throughout more than six months of treatment and follow-up, the patient’s rash and severe pruritus were well controlled ultimately achieving complete clinical remission (**Figure 4**). Objective assessments demonstrated a corresponding reduction in BPDAI from 120 to 0 and pruritus NRS from 9 to 0, while peripheral eosinophil counts returned to the normal range following bridging targeted therapy (**Table 1**; **Figure 3**).

## Discussion

In this case, the decision to initiate abrocitinib at 200 mg once daily was based on several clinical considerations. Standard BP treatment includes topical and oral corticosteroids, often combined with immunosuppressants (e.g., azathioprine, methotrexate, cyclosporine, mycophenolate mofetil) or immunomodulators (e.g., dapsone) (13). However, the most serious adverse effect of long-term immunosuppression is infection, which is also the most common cause of death in patients with bullous pemphigoid (14, 15). The 1-, 3-, and 5-year mortality rates were high, at approximately 26.7%, 45.7%, and 56.9%, respectively (14, 16). Notably, the first-year mortality risk was two to over seven times higher than that of the age- and sex-matched control group (17). Given the insufficient response to dose-escalated systemic corticosteroids, further glucocorticoid escalation was considered unlikely to provide additional benefit while potentially increasing treatment-related risks, whereas conventional immunosuppressive agents generally have a slower onset of action. Therefore, we have decided to introduce new targeted therapies targeting pathogenic immune pathways to improve treatment efficacy and reduce adverse events. However, in recent years, there has been no consensus on the management of BP (18). Because at treatment initiation, the patient presented predominantly with extensive erythematous patches and only a few scattered tiny blisters, whereas severe, rapidly progressive pruritus was the dominant clinical feature, resulting in an overall presentation resembling severe atopic dermatitis. Thus, abrocitinib was tried at a dose of 200 mg once daily as a transitional therapy to achieve rapid disease control. Considering that this is a 69-year-old female with hypertension who underwent lower limb fracture surgery a month ago, we conducted laboratory tests and thromboembolic risk assessment, and found no contraindications to short-term JAK1 inhibition in the patient.

As expected, the rapid relief of severe pruritus within 24 hours after initiation of abrocitinib, with the NRS decreasing from 9 to 4. No new blisters developed within six days. This rapid clinical response was considered particularly valuable. Because severe pruritus, a prominent feature of BP, occurs in nearly all patients (1). Uncontrolled pruritus not only substantially impairs quality of life but also perpetuates a self-perpetuating “itch–scratch–deterioration” loop, in which repeated scratching disrupts the skin barrier, causes skin erosions and exudation, increases the risk of secondary infection, and further aggravates disease severity. Although the precise mechanisms were not investigated in our patient, the rapid antipruritic effect of abrocitinib may be explained by its selective inhibition of JAK1-dependent cytokine signaling. Among these pathways, IL-31 is considered one of the principal mediators of pruritus in BP and signals predominantly through JAK1, making it a plausible therapeutic target (5, 19). In addition, inhibition of IL-4 and IL-13 signaling may contribute to attenuation of type 2 inflammation, thereby reducing disease activity (4). These mechanisms may collectively explain the rapid clinical improvement observed in our patient. Notably, transient fever and marked eosinophilia developed on the morning of day 3 after abrocitinib initiation. As the patient had only taken two doses of abrocitinib, the serum procalcitonin was normal, and blood culture was negative, an infectious cause of fever was considered unlikely. Given that high-dose intravenous methylprednisolone had been discontinued abruptly immediately before initiation of abrocitinib, the fever was considered most likely to represent a transient inflammatory response temporally associated with corticosteroid withdrawal.

Furthermore, dose reduction of abrocitinib from 200 mg to 100 mg daily promptly triggered relapse of pruritus and new tense bullae within one day. This may be explained by the fact that JAK inhibitors suppress cytokine signaling without eliminating pathogenic immune cell populations; consequently, reducing the degree of pathway inhibition may allow disease activity to recur rapidly (20). However, this interpretation remains speculative and requires confirmation in larger studies. Fortunately, disease control was re-established after the 200 mg dose was resumed. It should be acknowledged that, following relapse, thalidomide, Tripterygium wilfordii glycosides, promethazine, and ebastine were introduced concurrently with restoration of the 200 mg dose. Therefore, the subsequent clinical improvement cannot be attributed solely to abrocitinib. However, the immediate relapse with more severe symptoms after dose reduction, together with the rapid improvement previously observed during abrocitinib monotherapy, supports a substantial contribution of adequate JAK1 inhibition to disease control. Although these adjunctive immunosuppressants we used as salvage therapy may also have contributed to the subsequent improvement, as we know, these drugs have a relatively slow onset of action and a limited ability to suppress severe acute inflammatory activity. Thus, they are unlikely to be able to control such a rapidly progressive disease flare (21). In addition, after later stopping these immunosuppressants and keeping only abrocitinib and dupilumab, the patient’s condition remained stable, which can help us understand the baseline effect of targeted therapy. Moreover, Although multiple clinical trials, meta-analyses, and comprehensive safety analyses for skin diseases have confirmed the good safety profile of JAK inhibitors, abrocitinib still carries a boxed warning regarding serious infection, mortality, malignancy, major adverse cardiovascular events (MACE), and thrombosis (22–24). Therefore, patients should be carefully selected, dose-controlled, and closely monitored when using JAK inhibitors, despite the favorable clinical response observed in our patient.

Dupilumab was introduced after abrocitinib had achieved rapid disease control, primarily to maintain long-term remission while minimizing the risks associated with prolonged JAK1 inhibition. Dupilumab, an IL-4Rα antagonist, binds to the shared IL-4Rα subunit to simultaneously inhibit IL-4 and IL-13 signaling pathways, which are recognized contributors to type 2 inflammation in BP (25). Through these effects, dupilumab may reduce eosinophilic inflammation, suppress pathogenic immune responses, and improve skin barrier recovery, thereby supporting long-term disease control (25, 26). The recent FDA approval of dupilumab for moderate-to-severe BP provides disease-specific clinical evidence supporting its use in this setting (12, 27). In our case, complete clinical remission was maintained for four months after abrocitinib discontinuation while the patient received dupilumab monotherapy, without concomitant topical corticosteroids or other systemic immunosuppressive agents. Although conclusions cannot be drawn from a single case, this observation suggests that bridging transition from abrocitinib to dupilumab may represent a feasible maintenance strategy in selected patients with BP who require rapid initial disease control. Future studies are warranted to determine the optimal timing of transition, duration of bridging therapy, and patient selection for this potential bridging strategy.

## Limitations

Several limitations should be acknowledged. First, this is a single-case report, and the generalizability of the findings is inherently limited. Larger prospective studies are required to establish the long-term efficacy and safety of abrocitinib and dupilumab therapy. Second, the use of concomitant medications may confound interpretation of treatment effects. The rapid onset of symptom relief following initiation of abrocitinib and the relapse observed upon dose reduction support a primary role for JAK1 inhibition in the acute phase. However, the contribution of the adjunctive therapies cannot be completely excluded. Third, the combination regimen was used only in the short term under close monitoring and was transitioned to dupilumab monotherapy as soon as possible. VTE risk was assessed throughout this period and continued for 3 months after abrocitinib discontinuation. Nevertheless, the combination use of abrocitinib with dupilumab and other systemic drugs, including thalidomide and Tripterygium wilfordii glycosides, was an off-label approach, and evidence regarding its safety remains limited. Finally, mechanistic interpretations are based on known immunological pathways rather than direct biomarker measurements, and future studies incorporating longitudinal immune profiling are warranted.

## Conclusion

In conclusion, this case demonstrates that bridging therapy with abrocitinib followed by dupilumab achieved rapid disease control and sustained remission in a patient with bullous pemphigoid who showed an inadequate response to initial corticosteroid therapy. This approach offers a feasible bridging strategy that couples rapid induction with long-term maintenance in appropriately selected patients. While single-case findings require confirmation, this report provides a rationale for prospective evaluation of bridging targeted therapy in bullous pemphigoid.

## Data Availability

The original contributions presented in the study are included in the article/**Supplementary Material**. Further inquiries can be directed to the corresponding author.
